# Vaccine cold chain management practices in primary health centers providing an expanded immunization program in Northwest Ethiopia: self-reported and actual practice observational study

**DOI:** 10.3389/fpubh.2023.1194807

**Published:** 2023-07-27

**Authors:** Firdawek Shenkute Ergetie, Abebe Tarekegn Kassaw, Ashenafi Kibret Sendekie

**Affiliations:** ^1^Department of Pharmacy, Bahir Dar College of Health Sciences, Bahir Dar, Ethiopia; ^2^Department of Health Science, Gamby Medical and Business College, Bahir Dar, Ethiopia; ^3^Department of Pharmacy, College of Medicine and Health Science, Woldia University, Woldia, Ethiopia; ^4^Department of Clinical Pharmacy, School of Pharmacy, College of Medicine and Health Sciences, University of Gondar, Gondar, Ethiopia

**Keywords:** cold chain management, expanded program of immunization, health centers, practices, vaccines

## Abstract

**Background:**

Vaccines are vital health commodities that need an appropriate supply chain system. They could be transported, stored, and used at appropriate temperatures. The purpose of this study was to assess vaccine cold chain management practices in primary health centers offering an expanded program of immunization (EPI) in Bahir Dar, Northwest Ethiopia.

**Methods:**

A self-reported and actual practice observational cross-sectional study was conducted at primary public health centers in the Bahir Dar city administration from August 15 to 30, 2021. A simple random sampling method was used to select study participants. An interview-administered questionnaire and direct actual practice observation were used to collect data. The data was entered into the Epi-data 4.6 program and analyzed using SPSS version 25. Participants’ knowledge, storage and transport conditions, IPLS management, and vaccine cold chain management practices were examined using independent samples t-tests and one-way ANOVA tests.

**Findings:**

A total of 50 respondents from ten health centers were enrolled in the study. Most of the EPI service providers had good knowledge (60%) and good practice in vaccine storage and transport (74%). However, more than two-thirds (68%) of EPI service providers had poor integrated pharmaceutical logistics system (IPLS) management. Only half (50%) of the health centers had good actual vaccine cold chain management practices. Higher educational background, longer work experience, and receiving training and supervision in EPI services, IPLS management, and vaccine inventory management resulted in higher knowledge, storage and transport management, IPLS management, and vaccine cold chain management practices.

**Conclusion:**

Although most EPI service providers in an interview assessment reported having good knowledge and good vaccine storage and transport management, only half of the health facilities followed the standard. Stakeholders are recommended to play a vital role in improving practices related to EPI services.

## Introduction

Vaccines are sensitive to light, heat, and freezing, and they must be kept in a cold chain system within the World Health Organization’s (WHO) recommended temperature ranges (1, 2). All those who work with vaccines and diluents must be aware of the temperature sensitivities and recommended storage temperatures for all vaccines on the national schedule (3). A cold chain that meets specific temperature requirements is used to maintain vaccine quality. The cold chain is a network of storage and transportation links designed to keep vaccines at an acceptable temperature range from the manufacturer until they are delivered to users (4, 5).

From the time pharmaceuticals, vaccines, and other medicines are released from the manufacturer until they reach the final recipients, the entire supply chain system is expected to follow standardized procedures (6). These medications’ safety and efficacy are dependent on the system’s proper upkeep (7). The practice of vaccine cold chain management is continuing as one of the most critical initiatives that WHO has been promoting for more than 14 years. It includes vaccine storage practices, vaccine management personnel’s technical capacity, the logistics information system for vaccine commodities, and the distribution system. Based on these pillars, health facilities are expected to provide effective cold chain management practices and must be evaluated (8). The WHO also recommends that health facility refrigerators include a 30 day electronic refrigerator temperature logger and that facility staff be trained on how to use them. These devices record the refrigerator’s entire temperature history (3).

The success of the expanded immunization program (EPI) is thus heavily dependent on the state of the cold chain, and its management should not be taken lightly (9). Inadequate vaccine storage and management can decrease vaccine strength, resulting in poor vaccine-preventable diseases (VPDs) protection. In the fight against VPDs, proper vaccine storage and handling are critical. As a result, maintaining the vaccine’s potency and safety is critical in order to reduce childhood deaths from VPDs (4, 10, 11). Vaccines must be stored in good condition inside cold chain equipment with a low risk of temperature exposure (2). However, vaccine cold chain management has continued to be a difficult aspect of providing EPI services because of multiple challenges, particularly in underdeveloped settings (4, 10, 12, 13). Inadequate refrigerators, improper storage techniques, a lack of designated personnel, a lack of trained staff, issues with vaccine storage, a lack of temperature measurement devices, a lack of continuous temperature documentation, and other factors all contribute to the prevalence of these difficulties, especially at the lower levels of the health system. Similarly, the Clinton Health Access Initiative (CHAI) identified three major problems in the performance of cold chain management: a lack of cold chain capacity, a lack of modern technology or “optimal” equipment, and insufficient maintenance and temperature monitoring systems (12, 14). Other elements are related to the knowledge gaps among professionals who handle vaccines regarding vaccine waste stock management and control, monitoring, and distribution planning. The knowledge and capability of EPI service providers, such as training and supervision status, and the educational level of medical professionals can affect management practice (15, 16). As a result, providing training to EPI service providers significantly enhances cold chain management and ensures the potency and safety of vaccines (4, 13, 17), particularly in-service training (12, 18).

Ethiopia initiated EPI more than 50 years ago, but many of its citizens are still afflicted with diseases that can be prevented by vaccination. Out of every five children, one lacks immunity to diseases that can be avoided with vaccinations (19–22). The Ethiopian government has been working hard to increase vaccination coverage, and the quality of the immunization program is in line with GAVI’s strategy and the Sustainable Development Goals (SDGs) (23). The vaccination target phase-5 of the GAVI strategy for 2021–2025 seeks to place a far higher emphasis on reaching individuals who are most marginalized by enhancing primary healthcare systems, creating and sustaining community demand, and utilizing innovation to guarantee that these children receive immunization treatments.

Although effective vaccine and cold chain management play a prominent role in realizing quality provision of services, there are specific factors affecting the health sector in low-income countries such as Ethiopia that are not improving as planned (24–26). The absence of a constant and consistent power supply, a lack of cold chain system equipment, a lack of well-trained personnel with an excellent logistic system, and a lack of acceptable, adaptive, and self-updating policies and procedures are expected factors. Prior research in Addis Abeba, Ethiopia, for instance, reported that facilities generally employed appropriate cold chain management practices, but there were gaps in several areas, including storage conditions, transport trends, and awareness of EPI service providers (27, 28). However, they assessed only using interview-based self-reported methods, and the findings might not show the actual practice. As a result, the current study aimed to assess EPI providers’ knowledge, storage and transport conditions, IPLS management, and the actual cold chain management practices of primary health centers in Bahir Dar, Ethiopia, using both interview-based and actual practice observational assessment methods. We hope that this study provided comprehensive on the overall management practices of vaccines and the cold chain, as well as knowledge of EPI service providers.

## Methods and materials

### Study design and setting

A facility-based, mixed self-reported and actual practice-evaluating cross-sectional study was conducted at primary health centers providing EPI service in Bahir Dar city between August 15 and August 30, 2021. The study was conducted at government health centers in Bahir Dar’s city administration. Bahir Dar is located in the Amhara region, which is 568 kilometers northwest of Addis Ababa (the capital city of Ethiopia). The city is located in Northwestern Ethiopia; the altitude of Bahir Dar is between 648 and 1,300 meters above sea level, and it’s the third-largest city in Ethiopia. The city has an estimated population of over 400,000. In the city, there are ten government health centers providing EPI services.

### Study participants, eligibility, and sampling techniques

The study population for this study included EPI service providers who worked in the primary public health centers at Bahir Dar city administration. All EPI service providers who fulfilled the criteria were included. To be eligible for the study, they could be healthcare professionals who worked in the EPI service provision and in the vaccine and cold chain management systems. As a result, participants included EPI focal officers, vaccination providers, and vaccine and cold chain pharmacy store managers.

The participants were drawn from ten primary public health centers in the Bahir Dar city administration. According to Bahir Dar City Health Bureau and Bahir Dar Health Directive Department Officer’s data, there are a total of ten primary public health centers providing EPI service. Thus, because of the limited number of primary health centers in the city, we included all health centers that provided EPI services. The included health centers were: Abay, Bahir Dar, Han, Meshenti, Minilik II, Shimbit, Shumabo, Tiss-Abay, Zegie, and Zenzelima health centers.

A simple random sampling technique was used to select participants from the selected health centers using the lottery method proportionally among healthcare providers involved in the management system of the cold chain.

### Data collection instruments and procedures

A semi-structured questionnaire was developed by reviewing earlier studies. Mainly, two data collection methods were used to collect the data. Primary data was collected through face-to-face interviews using structured questionnaires. Secondly, the actual practices of vaccine and cold chain management at selected health facilities were evaluated by onsite observation with regard to their management practices of cold chain, storage conditions, logistic information systems, and availability of necessary equipment and logistics. The data collection instrument included four parts: the first consisted of participants’ sociodemographic characteristics. The second section consists of statements that describe the participants’ training and supervision, support from regulatory bodies, and knowledge-related questions. The third section of the instrument also contained eight statements used to assess vaccine storage and transport conditions and four items used for assessing IPLS management. The last section of the data collection was information used to directly observe the actual cold chain management practices of study participants in the selected health facilities using thirteen statements.

The internal validity of the instruments was checked, resulting in a Cronbach’s alpha of 0.84 for sixteen knowledge-related questions, 0.75 for eight questions used to assess storage and transport conditions, 0.72 for four IPLS questions, and 0.86 for thirteen questions that assessed the actual practice of vaccine management.

### Measurement of outcome variables

This study was focused on assessing the knowledge of participants, the storage and transport conditions of vaccines, integrated pharmaceutical logistic systems (IPLS), and the actual cold chain management practices of health facilities as outcome variables. Knowledge of the EPI service providers was assessed using 16 knowledge-related questions that described knowledge of how vaccines and diluents are stored at recommended temperatures, how to pack ice in the vaccine carrier, and the like, while storage and transport conditions of vaccines were assessed by interviewing EPI service providers using 8 vaccine storage and transport-related questions. Similarly, the vaccine IPLS management practice was assessed using four interview questions related to the transactions and IPLS management practices of vaccines in the selected health facility. On the other hand, the real practices of cold chain management at the selected health facilities were evaluated using 13 assessment questions related to vaccine and cold chain management practices through direct onsite observation of the health facilities.

All four variables were measured using “yes” and “no” questions with a respective score of 1 and 0, respectively. There could be a minimum score of 0 for all outcome variables and a maximum score of 16 for EPI service provider knowledge, 8 for vaccine storage and transport conditions and management, 4 for IPLS management, and 13 for the actual vaccine and cold chain management practice scores of the health facility. After computing the mean score using the sum score of all questions for each four outcome variables, participants who scored higher or equal to the mean knowledge related questions, vaccine storage and transport conditions questions, IPLS questions, and actual vaccine management practice questions were considered to have adequate knowledge, good vaccine storage and transport conditions, good IPLS management, and good vaccine and cold chain management practices, respectively and if they scored below the mean scores, they considered to have inadequate knowledge, poor vaccine storage and transport conditions, poor IPLS management, and poor vaccine and cold chain management practices, respectively (29).

### Data quality control measure

A pretest was conducted in two primary public health centers in Mecha woreda, Merawi town, to assess the quality of the data collection instrument. The samples for the pretest were selected using a simple random selection method among healthcare professionals involved in cold chain management. We selected ten professional healthcare providers from the health center. Based on the findings from the pretest, appropriate modifications were made to the data collection instruments so there was better data when finalizing the study.

The data collectors were two graduating pharmacy students who participated voluntarily. To ensure the quality of the data to be collected, the data collectors received appropriate training. They were trained on how to collect data for each question in the questionnaire for both interview and observational data collection items. Most importantly, for observational methods, the data collectors were practically trained through actual observation in the two health centers where the pretest was conducted. In addition, the supervisor was closely followed and intervened, particularly when there was variability in decision-making between the two data collectors during direct observation. Hence, he guided the data collectors to provide direction during data collection and was also able to check for completeness and quality. Furthermore, every filled-out interview and observation checklist was checked for its completeness, clarity, and cleanliness every day after data collection by the supervisor and data collectors.

### Statistical analysis and management

After the data is collected, it is entered into EPI-data version 4.6 and exported to SPSS version 25. A Q–Q plot and histogram were used to examine the data’s normal distribution. For categorical variables, descriptive statistics such as frequency and percentage were used, while continuous variables were presented using the mean with standard deviation. Furthermore, the final results collected using structured questionnaires and visual observation methods were summarized using tables. Independent sample *t*-tests and one-way ANOVA tests were used to examine the mean difference in knowledge of EPI providers, storage and transport conditions, IPLS management, and actual vaccine and cold chain management practices among the subgroups of the participants. A *p* value of <0.05 at a 95% level of significance was considered statistically significant.

## Results

### Sociodemographic characteristics of respondents

From the 50 enrolled study participants, 50 gave a complete response, making the response rate 100 percent. About more than half of the respondents (28, 56%) were female, with a mean age of 28.7 ± 5.7 years. Regarding their educational backgrounds, half (25, 50%) had a bachelor’s degree, while 15 (30%) and 10 (20%) were master’s degree and diploma holders, respectively. On the other hand, the majority (30, 60%) of respondents had 5–7 years of service experience, while others had an experience of less than or equal to four years and greater than or equal to eight years (10, 20%, for both), with an average of 7.2 (±3.6) years of experience.

### Knowledge of EPI service providers in cold chain management

According to an interview-based assessment, the majority of participants (60%) had a good level of knowledge on vaccine and cold chain management systems, with an overall mean score of 12.8 (±3.3) out of 16 possible points (ranges: 7–16). The majority of participants (88%) stated that they had received training on EPI services. Furthermore, the vast majority of participants (90%) reported receiving regulatory authority oversight. However, even if two-thirds (66%) of them reported that they were trained on IPLS management, more than half (52%) of the respondents reported that they were not trained on vaccine-related inventory management. Similarly, more than half (54%) of the EPI service providers failed to know the names of common light-sensitive vaccines. However, most participants claimed to be knowledgeable about the majority of the knowledge-related questions (Table 1).

**Table 1:** Training, supervision and technical capacity of EPI personnel in vaccine and cold chain management (*N* = 50).

Variables		Yes, *n* (%)
Training and supervision by regulatory body		
Trained on EPI service		44 (88)
Trained on IPLS training		33 (66)
Trained on vaccine related inventory management		24 (48)
Supervised by regulatory authorities within the last six months		45 (90)
Knowledge and technical capacity		
Knew the recommended temperature ranges for vaccine storage (+2°C and + 8°C).		47 (94)
Knew the recommended temperature ranges for diluents (cooled to 2–8°C).		41 (82)
Knew how to pack ice pack in vaccine carrier		34 (68)
Knew how to prevent vaccine freezing during transport		40 (80)
Knew most heat sensitive vaccines (OPV, Measles and BCG)		46 (92)
Named most cold sensitive vaccines (DPT-HepB-Hib, PCV, TT, IPV, and Rota)		41 (82)
Knew most light sensitive vaccines (BCG and measles)		23 (46)
Knew how to correctly conduct shake test		31 (62)
Knew vaccine vail monitor (VVM) stages		46 (92)
Knew vaccines eligible for four weeks open vial policy (TT, IPV and OPV)		39 (78)
Knew Early Expiry First Out (EEFO) principle based on VVM status		39 (78)
Knew Early Expiry First Out (EEFO) principle based on expiry date		48 (96)
Knew how to organize old and new vaccines to facilitate use of older vaccines first		40 (80)
Knew vaccines should be stored only for a maximum of 1 month in PHCF		45 (45)
Knew diluents and vaccine should be from same manufacturer		40 (80)
Knew how to calculate vaccines wastage rate		38 (76)
Overall mean score of knowledge		12.8(±3.3)
Level of knowledge of vaccine cold chain management	Poor	20 (40)
	Good	30 (60)

### Vaccine storage and transport conditions and logistic information systems

Almost three-fourths (74%) of the participants reported that storage and transport conditions of vaccines and the cold chain in their health center were good, with an overall mean score of 6.9 (±0.8) out of 8 (ranges: 4–8). Almost all (92%) respondents reported that their facility had a WHO-standard refrigerator. The majority of them (44, 88%) also reported that their facility had a standby generator to be used during an electric power failure. However, significant participants reported that their facility had no alternative storage (32%), lacked enough vaccine carriers (14%), and had no temperature record device during transportation (38%) (Table 2).

**Table 2:** Vaccine storage and transport conditions and logistic information systems (*N* = 50).

Variables		Yes, *n* (%)
Vaccine storage and transport conditions		
Availability of standard refrigerator (WHO standard)		46 (92)
Refrigerators with temperature alarm		46 (92)
Availability of standby generator		44 (88)
Vaccine FEFO usage		50 (100)
Availability of functional thermometer		49 (98)
Alternative storage		34 (68)
Enough vaccine carrier for transportation		43 (86)
Presence of temperature change record device during transportation		31 (62)
Overall mean score		6.9 ± (0.8)
Level of storage and transport conditions	Poor	13 (26)
	Good	37 (74)
IPLS management		
Vaccine supply recorded on receiving and issuing		50 (100)
Status of bin card updating regularly		29 (58)
Usage of VRF		48 (96)
Status of NO stock-out in the past 6 month		38 (76)
Overall mean score		3.3(±0.5)
Level of IPLS management	Poor	34 (68)
	Good	16 (32)

FEFO, first-expiry first-out; VRF, vaccine requisition forms.

Moreover, in terms of the facility’s logistic information system, overall, based on the respondent’s interview assessment, more than two-thirds (68%) of the respondents responded that their facility was poor in the logistic information system on vaccine and cold chain management, with a mean score of 3.3 (±0.5) out of 4 (ranges: 2–4). Only 29 (58%) of the respondents reported that they updated their bin cards regularly. However, all the respondents (100%) reported that their facilities received and dispatched vaccines using vaccine supply records and issued vouchers (Table 2).

### Observation of actual practices of vaccine and cold chain management system

During observation of the actual practices of selected health facilities, only half (50%) of the facilities were found to have good vaccine cold chain management practices, with an overall mean score of 7.2 (±0.9) out of 13 (ranges: 6–8). We observed that the majority of the facilities (60%) failed to obtain VVM posters and stickers, register all vaccines in the stock register, control the understock of vaccines in the last 6 months, and calculate the vaccine wastage rate monthly. Furthermore, only under one-third (30%) of health facilities updated their bin cards regularly with every transaction (Table 3).

**Table 3:** Observation of actual practice assessment of vaccine and cold chain management system at health facilities (*N* = 10).

Variables		Yes, *n* (%)
Refrigerator thermometer reading in NORMAL range during visit		9 (90)
Vaccines properly arranged within the refrigerator		7 (70)
Diluents properly packed inside the refrigerators		6 (60)
Ice packs properly packed inside the refrigerators		5 (50)
Did not keep expired vaccines in refrigerators		7 (70)
Updated record of temperature chart at least twice daily		6 (60)
Availability of VVM poster and sticker instructions		4 (40)
Updated bin card per the last transaction		3 (30)
Use standard vaccine requisition format for ordering and receiving vaccines		6 (60)
All vaccines properly registered in the stock register		4 (40)
Did not experience under-stock of any vaccine within the last six months		4 (40)
Did not experience over-stock of any vaccine within the last six months		7 (70)
Calculate vaccine wastage rate every month within the last six months		4 (40)
Overall mean score		7.2(±0.9)
Level of vaccine cold chain management practices	Poor	5 (50)
	Good	5 (50)

VVM, vaccine vail monitor.

### Knowledge, storage and transport conditions, IPLS management, and actual vaccine and cold chain management practice difference among subgroups

According to one-way ANOVA analysis, differences in educational status and work experience among participants resulted in significant differences in terms of knowledge, storage and transport conditions, IPLS management, and vaccine cold chain management. Further analysis of the Tukey *post hoc* test revealed that those with master’s degrees had significantly higher mean scores compared with diploma holders in terms of knowledge (*p* = 0.031), storage and transport conditions (*p* = 0.019), IPLS management (*p* = 0.032), and vaccine and cold chain management practice (*p* = 0.027). Professionals with work experience of greater than or equal to 8 years also had significantly higher mean scores compared with those who had less than or equal to four years’ experience in terms of knowledge (*p* = 0.017), storage and transport conditions (*p* = 0.033), logistic information system management (*p* = 0.021), and vaccine cold chain management practice (*p* = 0.015). An independent samples *t*-test showed that participants who were trained on EPI services, IPLS, vaccine inventory management, and were supervised by regulatory bodies, as well as those who had good knowledge, had significantly higher scores in terms of knowledge, storage and transport conditions, IPLS management, and vaccine cold chain management practices (Table 4).

**Table 4:** Knowledge, storage and transport, logistic information system, and vaccine cold chain management practice difference among sub groups of participants.

Variable		Knowledge of participants	Storage and transport condition	Logistic information system management	Vaccine cold chain management practice
		Mean (S ± D)	Mean(±SD)	Mean(±SD)	Mean(±SD)
Sex:	Male	13.0 (3.2)	6.7 (1.0)	3.1 (0.6)	6.9 (1.1)
	Female	12.6 (3.4)	7.1 (0.6)	3.5 (0.4)	7.5 (0.7)
	Value of *p*	0.342	0.541	0.183	0.095
Educational status	Diploma	10.8 (3.1)	6.5 (1.0)	2.72 (0.6)	6.2 (1.2)
	Bachelor’s degree	13.6 (3.5)	7.1 (0.8)	3.61 (0.5)	7.6 (1.0)
	Master’s degree	14.0 (3.3)	7.2 (0.6)	3.66 (0.4)	7.9 (0.6)
	Value of *p*	**0.013**	**0.025**	**<0.001**	**0.006**
Work experience	≤4	12.3 (3.4)	6.6 (1.1)	2.5 (0.7)	6.3 (1.1)
	5–7	12.9 (3.3)	7.0 (0.7)	3.6 (0.5)	7.5 (1.0)
	≥8	13.2 (3.2)	7.1 (0.6)	3.7 (0.3)	7.8 (0.7)
	Value of *p*	**0.032**	**0.041**	**0.027**	**0.016**
Trained on EPI service	Yes	13.5 (2.9)	7.6 (0.6)	4.1 (0.4)	8.3 (0.7)
	No	12.1 (3.7)	6.2 (1.0)	2.5 (0.6)	6.1 (1.1)
	Value of *p*	**<0.001**	**0.007**	**0.037**	**<0.001**
Trained on IPLS training	Yes	13.3 (3.1)	7.4 (0.7)	3.9 (0.3)	8.1 (0.6)
	No	12.3 (3.5)	6.0 (0.9)	2.7 (0.7)	6.3 (1.2)
	Value of *p*	**0.019**	**0.010**	**0.013**	**0.025**
Trained on vaccine inventory management	Yes	13.45 (3.0)	7.3 (0.5)	4.0 (0.35)	7.9 (0.8)
	No	12.05 (3.6)	6.5 (1.1)	2.6 (0.55)	6.5 (1.0)
	Value of *p*	**0.015**	**0.031**	**0.035**	**0.045**
Supervised by regulatory authorities	Yes	13.2 (2.9)	7.2 (0.7)	4.0 (0.3)	8.2 (0.6)
	No	12.4 (3.7)	6.6 (0.9)	2.6 (0.7)	6.2 (1.2)
	Value of *p*	**0.023**	**0.034**	**0.006**	**0.019**
Knowledge of EPI providers	Good	14.2 (3.1)	7.4 (0.6)	4.2 (0.4)	8.4 (0.7)
	Poor	11.4 (3.5)	6.2 (1.0)	2.4 (0.6)	6.0 (1.1)
	Value of *p*	**<0.001**	**<0.001**	**<0.001**	**<0.001**

Bold figures indicate, value of *p* < 0.05.

## Discussion

The current study highlighted the practice of vaccine and cold chain management using both interview-based and actual-practice observational studies. This study found that the majority (60%) of EPI service providers reported having good knowledge of the management of vaccines and the cold chain. Nearly three-quarters (74%) were also reported to have good storage and transport conditions. However, more than two-thirds (68%) of them reported having poor IPLS management. In terms of actual practice, only half (50%) of the health facilities were found to have good vaccine and cold chain management practices.

Knowledge of EPI service providers is critical for proper vaccine and cold chain management. In this study, in line with previous studies (19, 20), 60% of EPI service providers reported that they had an overall good knowledge score in vaccine and cold chain management. However, this finding is much higher than other findings from Ethiopia (29–32) and Nigeria (33, 34). The discrepancy might be due to differences in the sociodemographic characteristics of the participants regarding educational background, service experience, and access to guidance and training related to vaccine and cold chain management. For instance, the current study showed that significant participants were trained on EPI services and IPLS and accessed regulatory bodies’ supervision. As a result, EPI service providers need to enhance their capacity through training on the management of vaccines and the cold chain. They must also enhance their educational backgrounds through short-and long-term training.

The knowledge of EPI service providers regarding specific knowledge-related items was also assessed. The finding showed that the majority (>80%) of participants reported that they knew about the recommended vaccine and diluent’s storage temperature ranges. This finding is in line with earlier studies (32, 35). However, this finding was much higher than compared with other studies in Ethiopia (19, 20, 29–31, 36), Cameron (15), Mozambique (37), and Nigeria (38). Furthermore, in line with a study done in Malaysia (35), most of the EPI providers responded well to the shake test (62%), VVM stages (92%), heat-sensitive vaccines (92%), and freeze-sensitive vaccines (82%). These findings were much higher compared with earlier studies (15, 19, 29, 30, 32, 38, 39). In contrast, however, in this study, more than half (54%) of the EPI service providers failed to know the names of common light-sensitive vaccines. These discrepancies are possible because of differences in participants’ knowledge, work experience, and access to training and supervision.

The other important variable that this study assessed was the vaccine storage and transport management of EPI service providers. In this study, overall, around three-fourths of the EPI providers reported having good vaccine storage and transport trends in their facility. This finding is much higher than the results of the earlier studies conducted regarding vaccine storage and cold chain management practices (30, 36). The study also assessed specific items related to vaccine storage and transport conditions. Participants responded that more than 90% of vaccine refrigerators had functional thermometers with alarms. This finding is higher than that of a similar study conducted in the North Shoa region of Ethiopia (36). In addition, all healthcare providers were implementing the EEFO principle to store vaccines, which is higher than that of the study conducted in health facilities in east Gojjam, which was 43.3% (30). Regarding the presence of functional refrigerators, although the majority of the EPI service providers reported that their facility had a WHO-standard functional refrigerator, domestic nonstandard refrigerators have still been used among the selected health centers, which is against WHO recommendations (3). In fact, this figure was lower than a study by Cameron (40). In addition, even though these figures are better than earlier studies (30, 36, 40), a significant number of participants still reported that they did not have enough temperature change recording devices during transportation, alternative vaccine storage, or a vaccine carrier (container) for transportation. The finding may suggest that it could be an important task to monitor and record the temperature of vaccines throughout the supply chain process. This is the only way to ensure that vaccines have been kept in the right temperature range during storage. Therefore, these challenges need to improve to meet client demand for vaccine storage and transportation. Stakeholders and regulatory authorities are recommended to avail vaccine and cold chain storage and transportation materials and equipment as much as possible. Professionals working in EPI services are also expected to improve their skills and knowledge of vaccine storage and transportation conditions through short-and long-term training.

One of the mandatory roles of EPI service providers is vaccine IPLS management. It allows for the generation of accurate vaccine consumption data, refill requests, and the avoidance of vaccine waste (41). However, in contrast to other studies and the standards (28, 41), this study showed that the overall vaccine and cold chain IPLS management practice was low, with greater than two-thirds of the respondents reporting poor IPLS management. A higher percentage of participants also reported that they failed to update the status of bin cards and control stock management. The overstock and understock finding for vaccines was also higher than other studies reported in Ethiopia (29) and Cameron (15), but lower than a finding from South Africa (42). The discrepancies might be related to their IPLS and vaccine-related inventory management practices and experiences. This is because relatively fewer participants were trained on IPLS and vaccine-related inventory management, which are important for practicing proper IPLS management. This may relate to the fact that the participants in this study were professionals who were involved in EPI service delivery from multiple disciplines. However, trainings such as IPLS and stock inventory management commonly focus on pharmacy professionals. This study also revealed that professionals who were trained on IPLS and vaccine-related inventory management had good IPLS management. Therefore, it is better to provide IPLS and vaccine stock management training for all professionals who are engaged in EPI and cold chain management.

The other important area that this study demonstrated was the actual vaccine and cold chain management practices in the selected health facility. Though the majority of EPI service providers responded that they had good knowledge and experience of storage and transport conditions for vaccines and cold chain management, there were lots of gaps in the actual practice visits. Overall, half of the selected health facilities failed to practice vaccine and cold chain management according to the WHO’s recommendations. The majority of them failed to obtain VVM posters and stickers, register all vaccines in the stock register, control stock balances of vaccines, calculate vaccine wastage rates, and update their bin cards regularly. Even 30%–50% of the health centers did not store vaccines and diluents properly, did not pack ice packs properly inside the refrigerators, stored expired vaccines in the refrigerators, and did not update the record of the temperature chart at least twice daily. This might be because of the lack of commitment of EPI service providers towards proper vaccine and cold chain management practices and a lack of equipment and materials. However, the findings are comparable with those of earlier studies (19, 29, 30, 43). But this is much lower than other findings (20, 44, 45). The discrepancies might be because of differences in the availability of supplies and materials, as well as participant differences in terms of their knowledge, skills, and commitments. Therefore, improving skills and commitments might be valuable to enhance their practice. Lack of skills and poor knowledge may also contribute to this poor level of practice because around 40% of EPI providers who were involved in this study reported having poor overall knowledge. In this study, EPI service providers with more knowledge of vaccines and cold chain management practiced at a statistically higher rate than those with less knowledge. Therefore, stakeholders and regulatory bodies should have a better approach and support to improve actual vaccine and cold chain management practices by enhancing the capacity and knowledge of EPI service providers.

### Strength and limitations of the study

The self-reported data collection, which depends on the honesty and faith of the respondents, may affect the responses, resulting in an overestimation of current practice. The self-reported data collection method may result in recall bias. In addition, using a small sample size might make it difficult to draw conclusions and generalize. Despite these limitations, the study’s direct observation study design can show an actual practice and this study explored the extent of EPI service in primary healthcare settings through assessments of provider knowledge, storage and transport trends, IPLS management, and vaccine and cold chain management practices.

### Implication and public health contribution

Generally, this study presented a comprehensive finding regarding knowledge of EPI service providers, storage and transport conditions, IPLS management, and the actual management practice of vaccine and cold chain in primary health facilities. Although knowledge of most of the EPI service providers and storage and transport conditions was reported to be high in a self-reported assessment, there were still many challenges and gaps, particularly in the management of IPLS and actual management practices of vaccines and cold chains. Some of the challenges and gaps were a lack of materials and equipment for vaccine transport and storage, a lack of commitment to update temperature records at least twice daily, a failure to update bin cards per transaction, and a failure to manage stock status. We hope the findings of this study will help all stakeholder groups in this area, including policymakers, international organizations working on immunization, regulatory authorities, healthcare providers, and donors, in guideline development and implementation, compliance with minimum standards, and providing technical and financial support to public health facilities. This assessment also helps professionals understand how vaccines must be stored according to the guidelines set by the WHO. The results provide baseline data for any public health facility to take appropriate measures to improve the cold chain management practice of vaccines and for further studies.

## Conclusion

Overall, this study concluded that most of the EPI service providers in the study setting had good knowledge of vaccine storage and transport management. However, there is still a challenge with proper IPLS management regarding vaccine and cold chain management. Most importantly, actual vaccine and cold chain management practice in most of the health centers in the study setting was below the required standard. Educational background, service experience, and trainings related to EPI services of the EPI service providers played a role in their knowledge, vaccine storage and transport, IPLS management, and vaccine and cold chain management practices. As a result, stakeholders and regulatory bodies are encouraged to support and provide continuous supervision of EPI service providers’ actual vaccine and cold chain management practices.

## Data availability statement

The raw data supporting the conclusions of this article will be made available by the authors, without undue reservation.

## Ethics statement

The studies involving human participants were reviewed and approved by Gamby Medical and Business College’s Ethical Review Committee granted ethical approval (reference: GAMBC/428/2013). The patients/participants provided their written informed consent to participate in this study.

## Author contributions

FSE and ATK contributed to the conception, data curation, formal analysis, investigation, methodology, project administration, resources, and writing of the original draft and reviewed the final manuscript. AKS contributed to the conception, data curation, formal analysis, methodology, and validation, supervision, and reviewed the final manuscript. FSE, ATK, and AKS gave final approval of the version to be published, have agreed on the journal to which the article has been submitted and agreed to be accountable for all aspects of the work. AKS is the guarantor rater of this paper. All authors contributed to the article and approved the submitted version.

## Conflict of interest

The authors declare that the research was conducted in the absence of any commercial or financial relationships that could be construed as a potential conflict of interest.

## Publisher’s note

All claims expressed in this article are solely those of the authors and do not necessarily represent those of their affiliated organizations, or those of the publisher, the editors and the reviewers. Any product that may be evaluated in this article, or claim that may be made by its manufacturer, is not guaranteed or endorsed by the publisher.

## Abbreviations

CHAI: Clinton health access initiative
EPI: Expanded program on immunization
GAVI: Global alliance for vaccine initiative logistic information systems
PCV: Pneumococcal conjugate vaccine
VPDs: Vaccine preventable diseases
VVM: Vaccine vial monitor
WHO: World Health Organization
